# The Role of Rural Infrastructure in Reducing Production Costs and Promoting Resource-Conserving Agriculture

**DOI:** 10.3390/ijerph16183493

**Published:** 2019-09-19

**Authors:** Qinghua Wu, Xiaoliang Guan, Jun Zhang, Yang Xu

**Affiliations:** 1 School of Economics and Management, Wuhan University, Wuhan 430070, China; qhwu@whu.edu.cn; 2 College of Economics & Management, Huazhong Agricultural University, Wuhan 430070, China; guanxiaoliang@webmail.hzau.edu.cn; 3 School of Business Administration, Hubei University of Economics, Wuhan 430205, China; zhangjun200807@hbue.edu.cn; 4 School of Economics and Management, Hubei University of Science and Technology, Xianning 437100, China

**Keywords:** infrastructure, resource-conserving agriculture, production costs, unconditional quantile regression

## Abstract

The development of rural infrastructure plays an essential role in improving rural livelihoods and enhancing sustainable and environmentally friendly agricultural production. However, little is known about whether rural infrastructure enables the promotion of resource-conserving agriculture and reduces production costs. Understanding the relationship between rural infrastructure and production costs can provide significant information for policy-makers in their efforts to promote resource-saving agriculture that is beneficial to environmental performance. This study contributes to the literature by analyzing the heterogeneous effects of irrigation infrastructure and standard and substandard roads on agricultural production costs, using an unconditional quantile regression model and provincial data from China for the period 1995–2017. The empirical results show that the effects of rural infrastructure on production costs are mixed. In particular, irrigation infrastructure affects production costs positively in the lower quantiles, but it negatively affects production costs in the higher quantiles. In the higher 80th and 90th quantiles, standard and substandard roads affect production costs both negatively and significantly. Our findings suggest that improving rural infrastructure enables the promotion of resource-conserving agriculture and enhances environmental performance, especially for those paying high production costs.

## 1. Introduction

Although increasing input intensities and costs have largely boosted agricultural growth, they have posed a considerable threat to the sustainability of China’s agriculture. In particular, the negative externality of highly intensive chemical inputs has resulted in growing concerns regarding the environment and human health. According to the statistics of the Ministry of Agriculture and Rural Affairs of China in 2017, the effective utilization rates of fertilizers and pesticides among grain crops such as rice, wheat, and corn were only around 38%, [1]. Fertilizer overuse derived nitrogen leaching is one of the key causes of soil degradation and agricultural non-point source pollution. In addition, the excessive use of pesticides causes enormous health hazards for people. For the past few years, the side effects of pesticide residues on human health and environmental quality have been widely reported in China. This is partly because China’s agricultural support policies have significantly contributed to the increased use of agricultural fertilizers because, since 2004, they have encouraged farmers to enlarge farm size [2]. For example, the quantity of chemical fertilizer inputs was 46.37 million tons in 2004, but it has increased to 58.59 million tons in 2017 [3]. Furthermore, the amount of agricultural carbon emissions in China generally increased in that period, and the emissions from different categories of carbon sources uniformly show an increasing trend [4]. Thus, the development of resource-saving agriculture is proposed to improve the sustainability of agriculture through preventing environmental degradation [5,6].

Infrastructure is a fundamental factor that affects agricultural development, particularly in resisting natural disasters and reducing agricultural inputs. Generally, infrastructure comprises public utilities (such as power, telecommunications, piped water supply, sanitation and sewerage, solid waste collection and disposal, and piped gas), public works, and other transport sectors [7], with roads [8,9] and irrigation [9,10], including dams, canals, and ports, having a particular influence on agriculture, based on the practices in different areas. As all grown produce needs water and irrigation facilities, this directly affects the inputs and outputs of agriculture; however, the scarcity and unpredictability of the natural environment and the relationship between roads and transportation costs directly affect the marketing margin and also exacerbate market power by limiting farmers’ access to buyers [11]. Irrigation and road infrastructures can give rise to a substitutional effect or a complementary effect on labor, capital, and input factors in agricultural production. Therefore, in this article, we focus on irrigation and roads as the two most important kinds of infrastructures in rural areas.

The primary objective of this study is to investigate the relationship between infrastructure and agricultural production costs. Using provincial data for the period 1995–2017 and an unconditional quantile regression (UQR) model, we analyze the effects of irrigation and transport infrastructures on the costs of farming. The contributions of this study are twofold. Firstly, we construct an empirical model to take infrastructure into account in production functions based on the Public Expenditure Model and the Cost Function. Secondly, we employ an unconditional quantile regression model to investigate the effects of irrigation infrastructure, as well as standard and substandard roads, on agricultural production costs using the provincial data of China for the period 1995–2017. These results can provide empirical support to improve the competitive power of agricultural products and related policy efficiency.

## 2. Literature Review

In the development of economic growth theory, infrastructure is usually treated as an endogenous factor used to enhance economic performance. For example, infrastructure is fundamental to agricultural development under resource-constrained conditions, breaking the constraints of an unfavorable geographical location and local natural endowment [12]. It can also reduce agricultural production costs and trade costs by enhancing the viability of production alternatives [1,13]. However, the substitutability or complementarity of infrastructure determines whether it can be seen as a production factor or whether it influences agricultural production costs [7]. Barro proposed the Public Expenditure Model and treated infrastructure as a production factor, including it in the production function [14]. Afterwards, many scholars adopted Barro’s Public Expenditure Model on a theoretical basis. The model states that if one production factor is fixed, an increase in the input of public services and other factors of production will also increase the output of the production function. Moreover, the effect of infrastructure will inevitably cause the adjustment of the input structure of agricultural factors and the alteration of production costs.

In subsequent work, most studies have confirmed that infrastructure is an important factor that promotes the growth of the agricultural economy [1,8,15,16,17,18,19], but a wide variety of empirical outcomes using different kinds of data and approaches have also been obtained. Although irrigation facilities [15] and roads [8] are not normal production factors influencing the output, they are prerequisites of the rapid growth of an agricultural economy and directly or indirectly influence the inputs and marginal productivity of factors through different mechanisms of action. Roads play an important role in reducing transaction costs and improving the potential value of agricultural products, while irrigation facilities help reduce the costs of agricultural factors by decreasing the quality of the input and improving its efficiency [13]. For example, irrigation infrastructure helps to save the time needed for collecting water, and the freed-up time can then be reallocated in farm and nonfarm activities [20]. Irrigation infrastructure and the renovation of relevant institutions could provide security for irrigation and cash-crop development, even changing farmers’ agricultural input structure and costs to some extent. It is not appropriate to ignore infrastructure variables in the production function [21]. Some studies have investigated the relationship between infrastructure and agricultural production [9,22]. For example, Mamatzakis has reported that public infrastructure investment provides a significant return to agriculture and augments productivity growth, based on a model to decompose Greek agricultural productivity growth over the period 1960 to 1995 into components of technical change, returns to scale, and public infrastructure [9]. Alston and Pardey have stated that infrastructure is an important factor today that influences technology adoption, farm size, the mixture of outputs produced, and the structure of agricultural input [22]. However, different kinds of infrastructure completely differ from each other in physical characteristics and functions.

Irrigation and road infrastructure significantly affect agricultural production in many ways. Irrigation infrastructure has a heterogeneous effect according to natural character and farm size. For example, the construction of irrigation dams leads to a significant increase in the irrigated area and agricultural production and also provides insurance against rainfall shocks and temperature in the downstream districts, but the upstream areas do not have those benefits [20]. Better road condition helps decrease the transaction costs associated with agricultural activities. It can potentially reduce the costs of obtaining inputs and help to achieve much higher output prices, reducing the impact of local market shocks [13,23]. In particular, irrigation facilities and roads give rise to a substitution effect or a complementary effect on labor, capital, and other factors in agricultural production. For example, Mamatzakis has highlighted the fact that infrastructure, intermediate agricultural goods, and capital are complementary [9]. Teruel and Kuroda have intensively studied roads, irrigation, and electrical infrastructure and have found that roads and personal agricultural input are complementary, but irrigation facilities have the potential to reduce input costs through the substitutional effect on labor and intermediate inputs [17]. Presently, the profitability obtained from agricultural production for rural households is relatively low due to the persistent increased costs of production inputs. The increased expenditure on infrastructure by the government will partly lower natural and market risks in agricultural operation and will increase its profitability [1,18]. Zhang and Fan have demonstrated that irrigation facilities promote the outward shift of the agricultural production frontier and increase the productivity of crops [19]. Transport infrastructure reduces farmers’ input and transportation costs and increases the commercialization of agricultural products. However, little research has been done on the effects of irrigation and substandard and standard infrastructures on agricultural production costs, or on sustainable agricultural development in China.

## 3. The Theoretical Model and Empirical Approach

Agricultural production factors mainly include natural resources such as land, capital, labor, and technology. Only the combination of these production factors can achieve an agricultural production process. Technological efficiency partly determines the factor input quantity, and it influences the competitive power of agricultural products and the production costs of producers in the decision-making process. We assume that agricultural production outputs are a function of the production inputs (e.g., land, labor, and capital) and technology. Here, we can present the relationship between factor input and agricultural output as shown in Equation (1):

(1) Y=f(R,K,L,T)

where *Y*, *R*, *K*, *L*, and *T* are output, land, capital, labor, and technology, respectively. With the assumption that there is no crowding effect when using infrastructure, the Public Expenditure Model indicates that public services, including infrastructure, directly influence labor productivity and improve marginal production factors [14]. That view does not conflict with Gramlich and Duggal et al., who did not think of infrastructure as a kind of production factor because infrastructure is a factor directly affecting production [24,25]. Based on the Public Expenditure Model, this article incorporates infrastructure provided by all levels of government into the Cobb–Douglas product function. The agricultural production function can be expressed as follows:

(2) $$Y=AR^{\alpha }K^{\beta }L^{\delta }M^{\rho }G^{\delta }$$

where *A* is the total factor productivity (TFP), which represents the level of agricultural technology; 0 < *α*, *β*, *δ*, and *ρ* < 1. The sum of coefficients *α*, *β*, *δ*, and *ρ* reveal the condition of the scale economy in production. Equation (2) shows three potential situations regarding the effects of infrastructure on land (*R*), fixed capital (*K*), labor (*L*), intermediate factor input (*M*)*,* and infrastructure (*G*). These different conditions are increasing returns to scale, decreasing returns to scale, or constant returns to scale.

There are two important mechanisms by which infrastructure influences agriculture, and one of them has an effect on production costs, which can usually be expressed by the cost function. The cost function refers to the relationship between the costs (*C*), and output (*Y*), under the condition that the technical level represented by the TFP remains unchanged. After adopting the Household Contract Responsibility System in 1978, farmers handed over the country’s rural tax and the rural collective reserve funds, which can be seen as land rent, to the government or local village organization. In 2006, China completely cancelled the agricultural tax, so the land contractor did not need to pay land rent. Therefore, if we take capital (*K*), labor (*L*), and intermediate input product (*M*) into consideration, the cost function under the optimal condition is as follows:

(3) $$minC=K\cdot P_{K}+L\cdot P_{L}+M\cdot P_{M}$$

(4) $$ms.t\;Y=AK^{\beta }L^{\delta }G^{\delta }M^{\rho }$$

where *P_K_*, *P_L_*, and *P_M_* are the interest rate, wage, and the price of the intermediate input, respectively. Using the Lagrangian method to calculate Equations (3) and (4), we can obtain the following equation:

(5) $$C=3P_{K}\cdot {\left(\frac{P_{K}^{\beta }\cdot P_{L}^{\rho }\cdot Y}{A\cdot P_{L}^{\beta +\rho }\cdot G^{\delta }}\right)}^{\frac{1}{\beta +\delta +\rho }}.$$

From Equation (5), we know that the interest rate (*P_K_*), wage (*P_L_*), the price of intermediate input (*P_M_*), infrastructure (*G*), and technology (*A*) have a non-linear relationship with agricultural production costs (*C*). Taking the logarithm of Equation (5) and including the above three kinds of infrastructure, which are the irrigation infrastructure (IR), substandard road (*TR_1_*), and standard road (*TR_2_*), the following function can be obtained:

(6) $$lnC=\alpha_{0}+\beta_{1}lnIR+\beta_{2}lnTR_{1}+\beta_{3}lnTR_{2}+\beta_{4}lnP_{K}+\beta_{5}lnP_{L}+\beta_{6}lnP_{M}+\beta_{7}lnY$$

The price of production factors (*P_i_* (*i = K, L, M*)) and technological level (*A*) differ among provinces of China. The ordinary least square cannot accurately describe the influence of irrigation infrastructure (IR), standard roads (*TR_1_*), and substandard roads (*TR_2_*) on the variation range or the unconditional distribution shape of the agricultural costs (*C*). In terms of decision-making, the policies of random variables in different quantiles should also differ, especially when the economic level and government fiscal situations are different between the east, middle, and west of China. For example, the GDP per person in Beijing and Gansu was 21.1 thousand US dollars and 4.7 thousand US dollars (at the current price) in 2018, i.e., the first and last values in the comparison at the provincial level, separately. Therefore, the present study uses quantile regression to analyze the influence of infrastructure on agricultural production costs, as mentioned above. The quantile regression model can be expressed as Equation (7):

(7) $$lnC_{i,t}=\alpha_{i}+X_{i,t}\beta_{\theta }+\mu_{i,t},\;Q_{\theta }(lnC_{i,t}|X_{i,t},\beta_{\theta })=X_{i,t}\beta_{\theta }$$

where *X_i,t_* is the independent variable, $\alpha_{i}$ is the intercept term of the model, and *β_θ_* is the parameter vector. *Q_θ_*(*lnC_i,t_*|*X_i,t_*) is the unconditional quantile of *lnC_i,t_* when *X_i,t_* is given. The corresponding parameter vector of *β_θ_* in the unconditional quantile regression *θ* can be obtained by *X*, minimizing Equation (7), which can be described by Equation (8):

(8) $$min_{\alpha_{i}\left(\theta \right),\beta \left(\theta \right)}\left\{\underset{i}{\sum }\underset{t}{\sum }\theta |lnC_{i,t}-\alpha_{i}\left(\theta \right)-X_{i,t}\beta \left(\theta \right)\left|+\left(1-\theta \right)\right|lnC_{i,t}-\alpha_{i}\left(\theta \right)-X_{i,t}\beta \left(\theta \right)|\right\}$$

## 4. The Data and Descriptive Statistics

The proportion of agricultural production in the local GDP of Beijing, Tianjin, and Shanghai is less than 1 %, and the data in these three municipalities are incomplete (In 2017, the proportions of Beijing’s, Tianjin’s and Shanghai’s agricultural GDP were 0.9 percent, 0.9 percent and 0.4 percent, respectively. We do not take Taiwan, Hongkong, and Macao into account in this article). Thus, these three municipalities are excluded in our analysis, as is the province of Tibet. We primarily obtained data from 1995 to 2017 from the China Statistical Yearbook, compiled and issued by the National Bureau of Statistics, the Statistical Yearbooks from different provinces, and the China Agricultural Product Cost and Benefit Data Compilation (in the following, called The Data Compilation for short) [26]. The data from these sources mainly include agricultural inputs and their prices, agricultural GDP, the effective irrigation farmland of the substitution variable of irrigation infrastructure, standard roads, and substandard roads. However, the following instructions still need to be followed.

### 4.1. Agricultural Production Costs

The costs (*C*) are obtained by calculating the price and the amount of labor (*L*), fixed capital (*K*), and fertilizer (*M*), that is, *C = L × P_L_ + K × P_K_ + M × P_M_*. The China Statistical Yearbook does not provide investment data for agricultural fixed capital after 1999; we calculated this information by using the fixed capital investment and subtracting the completed investment of the buildings. Comparing the data with those before 1999, the statistical calibre can reach consensus. The employment data of the three industries at the provincial level do not appear in the second census of agriculture, so agricultural employment in 2006 in this article was taken from the Statistical Yearbooks of 2007 from different provinces (In fact, each province’s data for total employment and the main structured data are not strictly identical to the data in the China Statistical Yearbook as the data sources are different).The China Statistical Yearbook and the China Population and Employment Statistics Yearbook do not report provincial employment of the three industries after 2011, so agricultural employment after 2010 also came from the provincial Statistical Yearbooks. As the provincial statistical department originally collected the provincial data and then reported to the National Bureau of Statistics of China, we found that the data before and after 2011 are cohesive.

### 4.2. Labor Wage P_L_

The provincial wages are lower than the actual wages of hired labor. Considering that the price per hour differs in different places, the agricultural labor wages can be obtained by using the data in the Data Compilation (1996–2018), taking the price per day in each province and multiplying it by the number of working days. Thus, the provincial labor prices from 1995 to 2017 use the average labor wages per day for growing food and oil crops, cotton, hemp, tobacco, sugar-yielding crops, etc. Due to the lack of relevant statistical data, two problems should be highlighted. First, the costs and benefits, gathered according to the provincial labor price of each crop in the Data Compilation (1953–1997,1998), are absent, and so the hired labor wages from 1995 to 1997 cannot be obtained directly. This article, therefore, adopted the national industry-classified employees’ average wage increase rate as the provincial labor wage growth rate to determine the hired labor price in each area from 1995 to 1997. Secondly, the Data Compilation (2000) lacks data for labor wages in Ningxia and Qinghai provinces, and so we adopted the average labor wage of each crop in 1999 and 2000.

According to the China Rural Fixed Observation Survey Data, adding up the national per household labor inputs of the planting industry, forestry, animal husbandry, and the fishery industry can yield the agricultural laborers input per household. Then, dividing this by the amount of agricultural labor per household, we can obtain the labor inputs per capita from 2000 to 2002, which are 118.59 days, 115.48 days, and 110.56 days, respectively. However, after 2003, the statistics only include the labor input of crop farming and forestry. Combining the relevant research achievements and agricultural mechanization in China, the number of working hours per year per capita in this article in 2000 and before was 120 days, versus 105 days after 2000. Therefore, the labor wage (*P_L_*) is the product of the number of working days and the daily wage.

### 4.3. Fixed Capital Price P_K_

Capital price is usually expressed by the capital profit rate, which is equal to the ratio of the added economic value and the total capital. Here, we used each province’s production price index in agriculture and substituted it for the capital price. After the establishment of the Chongqing municipality in 1997, its price index of agricultural means of production is absent in the China Statistical Yearbook, so we used the data of Sichuan Province, which included Chongqing geographically before 1997.

### 4.4. Agricultural Intermediate Inputs Price, P_M_

Agricultural inputs mainly include fertilizers, seeds, agricultural films, pesticides, veterinary medicines, foodstuff, and feed additives. After 2007, the Data Compilation began to compute the quantity of the fertilizer input, including nitrogen, phosphate, potash, compound, and other fertilizers, per mu to be 666.67 square meters. The relevant data for most input products can rarely be found in the related yearbooks before 2007; however, we aimed to analyze the sustainable development of agriculture, and the overuse of fertilizers is the leading factor of water pollution in China as the economy booms [27]. In addition, cost of fertilizer accounts for the largest proportion of agricultural input expenditure. For example, it comprised 54.08% of material input costs of grain plants in China in 2017 [26]. The cost of fertilizers used by food and oil crops, cotton, hemp, tobacco, and sugar-yielding crops in The Data Compilation (1996 to 2018) is divided by fertilizer usage amount per hectare, so the arithmetic average and each province’s fertilizer price (*P_M_*) can be obtained by using the fertilizer cost per hectare, dividing its usage amount per hectar, and then multiplying this value by a thousand. However, the Data Compilation in 2002 does not include the crops’ usage amount of fertilizers per hectare in 2001. Therefore, this has been derived from the average data in 2000 and 2002 (The usage of fertilizers of some crops was not counted in 2001; and can be derived from the average numbers in 1999 and 2000).

There should be 713 samples for China’s 31 provinces (cities and autonomous regions), excluding Hong Kong, Macao, and the Taiwan regions, but the proportions of agricultural GDP in Beijing, Shanghai, and Tianjin are less than 1.0%, and the local governments provide a great deal of public resources for their agricultural development, making their operation greatly different from other provinces of China. There are also missing data for some years relating to substandard roads and irrigation infrastructure in Tibet and the abovementioned three areas. Furthermore, Chongqing was set up as a municipality directly under the central government in 1997, leading to missing data for that area in 1995 and 1996. Therefore, we obtained 619 samples at the provincial level between 1995 and 2017, and the descriptive statistics of each variable are shown in Table 1. With economic development and ageing, labor has been in relatively short supply since 2004, and the cost of labor keeps increasing, leading to the growth of agricultural production costs. At the same time, China’s government has invested a large amount of public resources into infrastructure construction, especially in the developed provinces, to help improve the conditions of irrigation and roads. For example, the effective irrigated area of farmland in Heilongjiang, one of the largest agricultural provinces, amounted to 6031.00 thousand hectares in 2017, and the extent of standard roads in Sichuan, located in the west of China, reached 294,809 kilometers. Meanwhile, since 2006, many substandard roads around the country have been improved to standard roads, with the highest extent of substandard roads being 109,429 kilometers in Hunan in 2006, after which the number in all provinces has continued to decrease. Owing to inflation, the indices of labor price, fixed capital price, input price, and agricultural GDP have increased significantly.

## 5. Empirical Results

Compared to mean-based approaches, such as ordinary least square (OLS) regression, quantile regression has apparent advantages. OLS regression can only be used to measure the explanatory variable’s average influence on the explained variable, *Y*’s conditional expectation, *E (l|x)*. In such cases, we might ignore the influence discrepancy of the explanatory variable *x* on the explained variable’s different conditional distributions. In contrast, quantile regression estimation can describe the explanatory variable’s influence on the explained variable more accurately, which is the quantile treatment effect (QTE) in the variation range and the adjustment of the distribution shape [28]. In addition, we can use the absolute value of the residual to minimize the objective function, reducing the effect of extreme values on coefficient estimation. Moreover, quantile regression can be divided into conditional quantile regression (CQR) and unconditional quantile regression (UQR), but the outcome of the former is greatly affected by individual heterogeneity. There are some shortcuts regarding the applicability and explanation of empirical outcomes because the conditional cumulative distribution function *F_l|x_*(·) strongly depends on the explanatory variable *x*, leading to the explained variable *y_τ_* of the conditional distribution *l|x* at quantile τ also being influenced by explanatory variable *x* [29].

The estimation approaches of unconditional quantile partial effects (UQPE) are based on the re-centered influence function (RIF) in the empirical part, the regression based on QTE and average treatment effect, and the general method assumes that the relationship between the explanatory variable *Y* and the explained variable *x* is linear. RIF is calculated as follows:

(9) $$\left(C_{i,t};q_{\tau },F_{C}\right)=q_{\tau }+\left[\left(\tau -\text{𝟙}\left\{C_{i,t}\leq q_{\tau }\right\}\right)/f_{C_{i,t}}\left(q_{\tau }\right)\right]$$

where *C_i,t_* is the dependent variable (agricultural production costs), τ is the specific quantile, *q_τ_* is the value of the dependent variable in the specific quantile, *F_Ci,t_* is the unconditional distribution function of *C_i,t_*, *f_Ci,t_* (*q_τ_*) is the density at the point *q_τ_*, and 𝟙{*C_i,t_* ≤ *q_τ_*} designates a dummy variable testing whether the dependent variable is less than *q_τ_*.

In the empirical process, the RIF is used as the dependent variable in the least square regression, as well as the change in the distribution of explanatory variables *X_i,t_* on quantiles of the unconditional marginal distribution of *C_i,t_*, which is shown in Table 2. As the RIF regression has a grid theory basis and accurately measures the marginal effect of the subtle change of a specific *x* on *Y* in quantile *τ*, we used it in empirical analysis and then compared it with the result of OLS regression.

The results in Table 2 show that in the different quantiles of production costs, infrastructure has two types of effect, which greatly differ from the results of conventional quantile regression (CQR) in the Table A1 of the Appendix A. The irrigation infrastructure (*IR*) and substandard roads (*TR_2_*) only reduce the costs in high quantiles; the elasticity of irrigation that is the most foundational agricultural facility is −1.092 in the 80th quantile, and the elasticity of substandard roads is −0.199. This is partly because those two types of infrastructure are frequently used in high proportion agricultural GDP provinces. For example, according to the China Statistical Yearbook of 2018, the agricultural GDP values of Shangdong, Sichuan, Henan, and Jiangsu were the four highest among the provinces in 2017, and irrigation condition in those areas was, on the whole, also better than in other areas [3]. This leads to the operator facing more costs and thus provides stronger incentives for peasants to make good use of the infrastructure. However, the government tends to invest in large and mid-sized irrigation infrastructures such as reservoirs, big rivers, and lakes, and the construction and maintenance of small facilities primarily depends on local governments, village collectives, and residents. We used relevant data from the China Statistical Yearbook (2018) to determine that the number of large-, mid-, and small-sized reservoirs had increased by 1.74 times, 1.45 times, and 1.17 times, respectively, compared to 2000 [3]. As a result, a small agricultural operator is less likely to input many resources, leading to an increase in the importance of irrigation infrastructure, which is strongly related to agricultural production. Although the substandard roads in China have formed an excellent system, they merely help the agricultural products reach places over a shorter distance, and the effect of saving transportation costs is comparatively smaller than that of other infrastructures.

**Figure 1:**
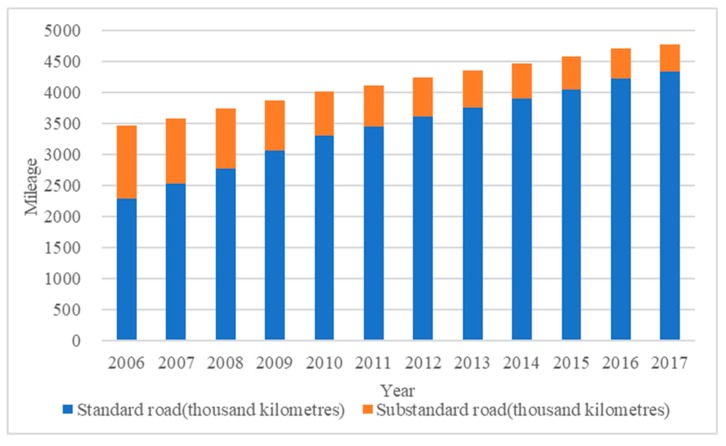
The Development of China’s Roads, 2006 to 2017. Source: China Statistical Yearbook (2007–2018) [3].

Standard roads (*TR_1_*) have a negative correlation coefficient between −0.218 and −0.443, which means that they exhibit a cost-saving effect largely attributed to their condition and physical properties, in contrast to the other two kinds of infrastructure. Transportation systems are strongly correlated with the level of economic development because they can lower transport costs, speed up specialization, and improve agricultural productivity [13]. However, we need to take the condition of industrial structure and transportation into account. Since the 21st century, China’s standard roads, especially highways, have developed quickly, and the proportion of substandard road has become much smaller (see Figure 1). In 2006, the extent of standard and substandard roads was 2282.87 thousand kilometres and 1174.13 thousand kilometres, respectively, whereas in 2017 the values were 4338.56 thousand kilometres and 434.91 thousand kilometres, with some improvement of substandard roads [3]. It is known that standard roads have many advantages in terms of traffic volume and speed, and we can achieve economies of scale as the stock of standard road grows. Therefore, standard roads greatly reduce trade costs and further promote the circulation of products [30], leading to their much more critical role in the logistical process.

With regard to the labor price, empirical outcomes show that increased labor price (*P_L_*) is the most important factor affecting production costs, and the fixed capital price has an unstable effect. Taking the average labor price of China’s three staple crops (rice, wheat, corn) from 1998 to 2017 as an example, cost of labor has sharply increased since 2002. In the same period, the lowest agricultural wage per day was 14.05 Yuan in 1999, after which it continued to increase, reaching 116.47 Yuan in 2017; i.e., it had tripled, while production costs had little more than doubled, increasing to 866.01 Yuan in 2017 from 321.15 Yuan in 1999 [26]. Moreover, we can obtain some evidence from changes in industrial structure and wages among those industries.

Fixed Capital Price (*P_K_*) has an inverted U curve effect, which means it has a positive effect in the low quantile, but the effect changes to negative in the high quantile. With the increase of production costs, the fixed capital input decreases sharply; the elasticity of fixed capital to costs is –5.565 in the 90th quantile, and it doubles with the elasticity in the 70th quantile. As we know, there is a specific threshold value, which is mainly decided by farmland endowment and the varieties of crops or animals being farmed. However, agriculture has the feature of low value yields compared with non-agricultural industries. When the potential investment into agricultural production increases, the operator tends to take his capital into other fields instead of agriculture because agricultural operation is often faced with a much higher risk from nature and the markets than other industries, and the household farmland scale is still very small in China. The farmer cannot generally gain from scale economy, measured by the index of arable land per person, and the proportion of agricultural employment is much higher than that in most developed countries. Based on the data of the World Bank Database, the proportion of employment supplied by agriculture was 26.98%, which almost correlates with Chinese governmental data (27.0% in 2017) and is much higher than that of some countries, such as Brazil (9.50%), Canada (1.52%), Japan (3.44%), and the United States (1.43%) [31]. Thus, the opportunity cost of the labor increase will lead to an increasing input trend at the provincial level.

**Figure 2:**
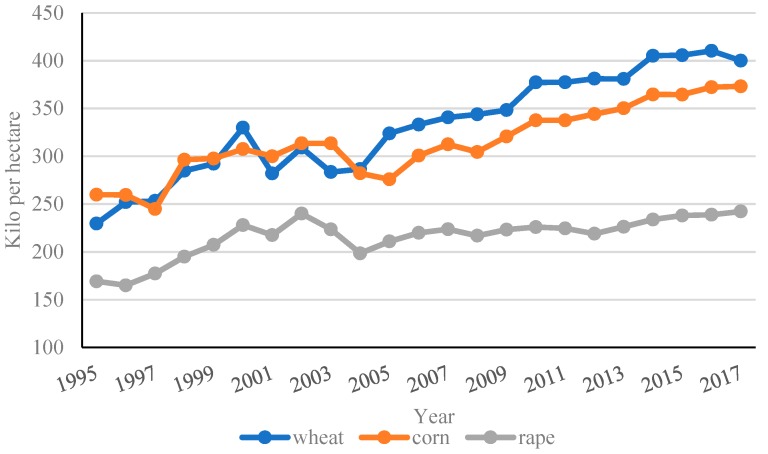
Chemical fertilizer input of widely planted crops in China, 1995 to 2017.

In addition, the quality of China’s fertilizer input in a unit area partly leads to the cost-saving effects of infrastructure on cost not being significant because the input is much higher than that in the other main areas of the world. According to the data of the World Bank, China’s fertilizer consumption was 377.46 kilograms per hectare of arable land in 2002, and the average global number was only 107.60 kilograms for the same period. In 2016, consumption in China increased to 503.12 kilograms after all levels of governments took steps to reduce the agricultural use of chemicals, but the consumption values globally and in the European Union, North America, South Asia, Latin America, and the Caribbean were recorded as 140.55, 158.38, 127.22, 160.28, and 140.19, respectively [32]. We took three kinds of China’s widely planted crops as examples (wheat, corn, and rape) and plotted the trend of fertilizer inputs in Figure 2. The figure shows that the amounts of fertilizer inputs of these three crops fluctuated before 2005 and experienced a continuous increase after 2005. The most remarkable increase happened in wheat, and the fertilizer usage of corn and rape also increased in the same period. The average fertilizer input of wheat in China was 400.05 kilograms per hectare in 2017, which was nearly two times the quantity used in 1995 [3]. Furthermore, in 2017, the usage in a hectare of corn and rape reached 373.20 kilograms and 242.25 kilograms, separately, and the yearly growth rate was about 1.6% for those plants [26]. This phenomenon is partly due to a reduction in the comparative price of fertilizer, with increasing wages after 2003. To gain high output, the quantity of fertilizer used increased year by year, with cultivated land quality declining, which directly induced environmental degradation, resulting in a formidable challenge for agricultural development.

## 6. Conclusions

The development of rural infrastructure plays an essential role in improving rural livelihoods and enhancing sustainable and environmentally-friendly agricultural production. For example, infrastructure significantly affects socioeconomic outcomes and agricultural development patterns [18,33]. However, little is known about whether rural infrastructure enables the promotion of resource-conserving agriculture and reduces production costs. Based on the Public Expenditure Model and agricultural production function, this study includes irrigation infrastructures and substandard and standard roads in the agricultural production function to examine their effects on agricultural production costs, using an unconditional quantile regression model and provincial data from China between 1995 and 2017. The results show that standard roads have a negative effect on China’s agricultural production costs. The effects of irrigation infrastructure and substandard roads on production costs are negative when the costs are high but are positive when the costs are small, and the quality of China’s fertilizer input in a unit area partly leads to the cost-saving effects of infrastructures on cost not being significant.

Our findings have important policy implications for resource-conserving agricultural development that actually promote environmental sustainability. The discovery of a negative and statistically significant effect of irrigation and substandard roads on agricultural production costs highlights the fact that the government needs to pay attention to the construction and maintenance of irrigation and road infrastructures and to encourage farmers to increase their operation scale by using measures of farm-based vocational training and agricultural mechanization subsidies.

Agricultural costs are only one aspect of infrastructure’s influence on agriculture, and some fields still need further study. Firstly, due to the lack of household data, this article does not analyze the main influence of infrastructure on the costs of micro-producers. Secondly, rural transport facilities lessen the effects of geographical location by making it easier to get goods from the place of production to the place of consumption and also effect the selection of agricultural location [13]. Farmers’ motivation for profit promotes the institutional change of the construction of irrigation infrastructure and causes a change in planting structure. Further study is needed to determine the influence of infrastructure on planting structure in the future. Thirdly, profit is the lifeline of the sustainable development of agriculture, which is codetermined by cost and profit, but the influence of infrastructure on agricultural profits is not included in this article. Furthermore, as infrastructure can be divided into different categories, analyzing its influence on the production of different industries from a microcosmic perspective can reduce the risk of omitting important variables.

## Figures and Tables

**Table 1:** Descriptive statistics of each variable.

Variable	Unit	Mean	SD	Maximum	Minimum
Agricultural production costs (*C*)	billion Yuan	78.06	79.20	535.06	2.03
Effective irrigated area (*IR*)	thousand hectares	2105.75	1413.01	6031.00	168.27
Standard road (*TR_1_*)	km	86,299.17	60,716.63	294,809.00	7912.00
Substandard road (*TR_2_*)	km	17,958.56	19,497.58	109,429.00	0.00
Labor price (*P_L_*)	thousand Yuan per capita	4.83	3.47	14.52	0.89
Fixed capital price (*P_K_*)	--	1.04	0.08	1.36	0.90
Input price (*P_M_*)	thousand Yuan per ton	4.57	1.32	8.01	2.25
Agricultural GDP(*Y*)	Billion Yuan	127.28	121.26	914.04	3.85
Note: SD = standard deviation.					

Notes: In the China Statistical Yearbook (1996), road mileage is subdivided into paved highways and non-paved highways. Here, substandard roads in 1995 are substituted by non-paved highways.

**Table 2:** Results of unconditional quantile estimation and ordinary least square (OLS) regression.

Variables	Q10	Q20	Q30	Q40	Q50	Q60	Q70	Q80	Q90	OLS
*lnIR*	0.886 ^*^ (0.464)	1.036 ^***^ (0.395)	0.965 ^***^ (0.254)	0.346 ^*^ (0.200)	−0.075 (0.222)	−0.295 (0.285)	−1.056 ^***^ (0.370)	−1.092 ^**^ (0.463)	−0.656 (0.671)	0.099 ^***^ (0.034)
*lnTR_1_*	0.124 (0.202)	−0.233 ^**^ (0.119)	−0.061 (0.118)	−0.133 (0.103)	0.074 (0.092)	−0.120 (0.125)	−0.218 ^*^ (0.124)	−0.443 ^**^ (0.193)	−0.362 ^*^ (0.212)	0.167 ^***^ (0.037)
*lnTR_2_*	−0.118 ^*^ (0.067)	0.061 (0.066)	0.134 ^***^ (0.048)	0.187 ^***^ (0.041)	0.068 ^*^ (0.036)	0.025 (0.046)	0.093 ^*^ (0.053)	−0.075 (0.071)	−0.199 ^***^ (0.071)	0.036 ^***^ (0.009)
*lnP_L_*	0.914 ^***^ (0.333)	0.817 ^***^ (0.211)	0.579 ^***^ (0.207)	0.617 ^***^ (0.216)	0.814 ^***^ (0.230)	1.569 ^***^ (0.229)	1.912 ^***^ (0.299)	1.179 ^***^ (0.340)	0.297 (0.492)	0.472 ^***^ (0.054)
*lnP_K_*	0.607 (1.720)	0.254 (1.127)	0.245 (0.786)	−1.371 (0.880)	−0.288 (0.820)	0.480 (0.891)	−2.565 ^*^ (1.519)	−4.366 ^***^ (1.252)	−5.565 ^***^ (1.359)	−1.843 ^***^ (0.439)
*lnP_M_*	−0.701 (0.442)	−0.118 (0.310)	0.047 (0.192)	−0.048 (0.204)	0.014 (0.189)	0.257 (0.213)	0.291 (0.371)	0.259 (0.373)	0.580 (0.390)	−0.098 (0.112)
*lnY*	−0.171 (0.273)	−0.012 (0.151)	0.010 (0.153)	0.258 (0.162)	0.232 (0.175)	0.066 (0.185)	0.475 (0.300)	1.109 ^***^ (0.273)	1.349 ^***^ (0.479)	0.474 ^***^ (0.039)
Constant	−3.935 (3.210)	−3.600 (3.557)	−5.371 ^**^ (2.457)	−0.170 (1.835)	0.837 (1.964)	3.816 (2.389)	9.956 ^***^ (3.020)	14.325 ^***^ (3.726)	12.444 ^***^ (4.646)	−0.317 (0.432)
*R* ^2^	0.126	0.258	0.369	0.449	0.518	0.593	0.649	0.517	0.306	0.862
*Observation*	619	619	619	619	619	619	619	619	619	619

Note: Standard errors in parentheses; * *p* < 0.1, ** *p* < 0.05, *** *p* < 0.01.

## Appendix A

**Table A1:** Empirical results; conventional quantile regression (CQR).

	(1)	(2)	(3)	(4)	(5)	(6)	(7)	(8)	(9)
Variables	Q10	Q20	Q30	Q40	Q50	Q60	Q70	Q80	Q90
*lnIR*	0.631 ^***^ (0.053)	0.577 ^***^ (0.057)	0.484 ^***^ (0.047)	0.488 ^***^ (0.038)	0.511 ^***^ (0.038)	0.477 ^***^ (0.036)	0.422 ^***^ (0.034)	0.411 ^***^ (0.041)	0.420 ^***^ (0.055)
*lnTR_1_*	0.004 (0.051)	0.033 (0.055)	0.132 ^***^ (0.046)	0.143 ^***^ (0.037)	0.111 ^***^ (0.037)	0.152 ^***^ (0.034)	0.189 ^***^ (0.033)	0.182 ^***^ (0.040)	0.138 ^***^ (0.053)
*lnTR_2_*	0.228 ^***^ (0.062)	0.250 ^***^ (0.066)	0.178 ^***^ (0.056)	0.150 ^***^ (0.045)	0.142 ^***^ (0.045)	0.123 ^***^ (0.042)	0.138 ^***^ (0.040)	0.070 (0.048)	0.059 (0.064)
*lnP_L_*	0.019 (0.017)	0.039 ^**^ (0.019)	0.037 ^**^ (0.016)	0.049 ^***^ (0.012)	0.038 ^***^ (0.012)	0.049 ^***^ (0.012)	0.044 ^***^ (0.011)	0.036 ^***^ (0.014)	0.036 ^**^ (0.018)
*lnP_K_*	0.279 ^***^ (0.080)	0.323 ^***^ (0.086)	0.447 ^***^ (0.072)	0.490 ^***^ (0.058)	0.507 ^***^ (0.058)	0.564 ^***^ (0.054)	0.569 ^***^ (0.052)	0.630 ^***^ (0.062)	0.643 ^***^ (0.083)
*lnP_M_*	−1.492 ^**^ (0.647)	−2.294 ^***^ (0.694)	−2.009 ^***^ (0.581)	−1.861 ^***^ (0.468)	−1.837 ^***^ (0.467)	−1.643 ^***^ (0.435)	−1.486 ^***^ (0.423)	−1.554 ^***^ (0.506)	−1.595 ^**^ (0.672)
*lnY*	−0.214 (0.162)	−0.202 (0.174)	−0.079 (0.145)	−0.122 (0.117)	−0.208 ^*^ (0.117)	−0.161 (0.109)	−0.092 (0.106)	−0.049 (0.127)	−0.037 (0.168)
Constant	−1.045 (0.679)	−0.793 (0.729)	−0.745 (0.609)	−0.677 (0.491)	−0.174 (0.490)	−0.442 (0.457)	−0.754 ^*^ (0.443)	0.141 (0.531)	0.691 (0.705)
*R* ^2^	0.631	0.617	0.620	0.630	0.642	0.653	0.665	0.664	0.653
Observations	619	619	619	619	619	619	619	619	619

Notes: Standard errors in parentheses; ^*^ *p* < 0.1, ^**^ *p* < 0.05, ^***^ *p* < 0.01.
